# Dihydropyrimidinase Like 2 Promotes Bladder Cancer Progression via Pyruvate Kinase M2-Induced Aerobic Glycolysis and Epithelial–Mesenchymal Transition

**DOI:** 10.3389/fcell.2021.641432

**Published:** 2021-07-06

**Authors:** Jun Zou, Ruiyan Huang, Yanfei Chen, Xiaoping Huang, Huajun Li, Peng Liang, Shan Chen

**Affiliations:** ^1^Department of Emergency Surgery, The Third Affiliated Hospital of Guangzhou Medical University, Guangzhou, China; ^2^State Key Laboratory of Oncology in South China, Department of Ultrasonography and Electrocardiograms, Collaborative Innovation Center for Cancer Medicine, Sun Yat-sen University Cancer Center, Guangzhou, China; ^3^Department of Urology, Affiliated Cancer Hospital and Institute of Guangzhou Medical University, Guangzhou, China

**Keywords:** epithelial–mesenchymal transition, M2-type pyruvate kinase, dihydropyrimidinase like 2, bladder cancer, aerobic glycolysis

## Abstract

**Background:**

Aerobic glycolysis and epidermal–mesenchymal transition (EMT) play key roles in the development of bladder cancer. This study aimed to investigate the function and the underlying mechanism of dihydropyrimidinase like 2 (DPYSL2) in bladder cancer progression.

**Methods:**

The expression pattern of DPYSL2 in bladder cancer and the correlation of DPYSL2 expression with clinicopathological characteristics of bladder cancer patients were analyzed using the data from different databases and tissue microarray. Gain- and loss-of-function assays were performed to explore the role of DPYSL2 in bladder cancer progression *in vitro* and in mice. Proteomic analysis was performed to identify the interacting partner of DPYSL2 in bladder cancer cells.

**Findings:**

The results showed that DPYSL2 expression was upregulated in bladder cancer tissue compared with adjacent normal bladder tissue and in more aggressive cancer stages compared with lower stages. DPYSL2 promoted malignant behavior of bladder cancer cells *in vitro*, as well as tumor growth and distant metastasis in mice. Mechanistically, DPYSL2 interacted with pyruvate kinase M2 (PKM2) and promoted the conversion of PKM2 tetramers to PKM2 dimers. Knockdown of PKM2 completely blocked DPYSL2-induced enhancement of the malignant behavior, glucose uptake, lactic acid production, and epithelial–mesenchymal transition in bladder cancer cells.

**Interpretation:**

In conclusion, the results suggest that DPYSL2 promotes aerobic glycolysis and EMT in bladder cancer via PKM2, serving as a potential therapeutic target for bladder cancer treatment.

## Research in Context

Aerobic glycolysis and epidermal–mesenchymal transition (EMT) play key roles in the development of bladder cancer. However, the underlying mechanism remains largely unknown. It was shown that DPYSL2 expression was upregulated in bladder cancer tissue compared with adjacent normal bladder tissue and in more aggressive cancer stages compared with lower stages. DPYSL2 promoted malignant behavior of bladder cancer cells *in vitro*, as well as tumor growth and distant metastasis in mice. Mechanistically, DPYSL2 interacted with pyruvate kinase M2 (PKM2) and promoted the conversion of PKM2 tetramers to PKM2 dimers. Knockdown of PKM2 completely blocked DPYSL2-induced enhancement of the malignant behavior, glucose uptake, lactic acid production, and epithelial–mesenchymal transition in bladder cancer cells. In conclusion, the results suggest that DPYSL2 promotes aerobic glycolysis and EMT in bladder cancer via PKM2, serving as a potential therapeutic target for bladder cancer treatment.

## Introduction

Despite recent diagnostic and therapeutic advances, the incidence and mortality of bladder cancer are increasing worldwide. In 2012, 430,000 new cases of bladder cancer have been reported worldwide, with 160,000 deaths (Antoni et al., 2017); in 2018, the number of new cases has increased to 549,393, with 199,922 deaths (Bladder Source Globocan, 2018). It is urgent to reveal the mechanisms underlying bladder cancer progression and to find new therapeutic targets for bladder cancer treatment.

Epithelial–mesenchymal transition (EMT) is closely related to cancer progression by enhancing cancer motility and dissemination through the disruption of intercellular junctions (Nieto et al., 2016). The loss of epithelial marker E-cadherin and the gain of mesenchymal markers, such as N-cadherin, vimentin, and ZEB1/2, are key events in EMT (Liao and Yang, 2017; Rout-Pitt et al., 2018; Aiello and Kang, 2019; Mrkvicova et al., 2019). It is also known that cancer cells exhibit aerobic glycolysis or the Warburg effect for energy production during proliferation (Warburg, 1956; Zhang et al., 2019). Studies have shown that both EMT and aerobic glycolysis contribute to tumor progression and metastasis in bladder cancer (Ritterson Lew et al., 2015; Islam et al., 2016); however, the regulators that trigger EMT and aerobic glycolysis in bladder cancer remain largely unknown.

Pyruvate kinase M2 (PKM2) catalyzes the final and rate-limiting reaction of glycolysis, playing a key role in aerobic glycolysis in cancer cells (Mayumi et al., 2012; Chen et al., 2019; Zhang et al., 2019). PKM2 switches between a highly active tetramer and a lowly active dimer in healthy cells but tends to exist as a dimer in cancer cells. An increased level of dimeric PKM2 shifts the glucose metabolism from the normal respiratory pathway (TCA cycle) to aerobic glycolysis (lactate production) in cancer cells, promoting cancer cell proliferation and growth (Li et al., 2014; Israelsen and Vander Heiden, 2015). PKM2 also has been shown to promote EMT in colon cancer cells by functioning as a dimeric protein kinase and inhibiting E-cadherin expression upon translocation into the nucleus (Hamabe et al., 2014). Thus, targeting dimeric PKM2 may be a potential therapeutic strategy for cancer treatment, and it is critical to identify the molecules that control the switching between the dimeric and tetrameric PKM2.

Dihydropyrimidinase-related protein 2 (DPYSL2), also known as collapsin response mediator protein 2 (CRMP2), belongs to the CRMP family that shares about 50–70% sequence homology, promoting tumor progression in multiple cancer types, including gastric cancer (Yutaka et al., 2013), colon cancer (Goulet et al., 2007), and breast cancer (Tominaga et al., 2019). However, the involvement of DPYSL2 in bladder cancer progression remains unknown. Studies have shown that DPYSL isoforms, such as DPYSL1 and DPYSL3, modulate EMT in cancer (Cai et al., 2017; Matsunuma et al., 2018). In addition, DPYSL2 is implicated in glucose metabolism. Knockdown of DPYSL2 reduces insulin-induced glucose uptake in 3T3-L1 adipocytes (Chang et al., 2020). DPYSL2 is highly expressed in the pancreatic islet, participating in the pathogenesis of type 2 diabetes (Nicolls et al., 2003). However, whether DPYSL2 regulates EMT and aerobic glycolysis in cancer remains unexplored.

In this study, we aimed to investigate the role of DPYSL2 and to identify its interacting partner in EMT and aerobic glycolysis during bladder cancer progression. The expression pattern of DPYSL2 was compared between non-muscular invasive bladder cancer (NMIBC) and more aggressive muscular invasive bladder cancer (MIBC) by analyzing the patient data from different databases, as well as between bladder cancer tissue and adjacent non-cancerous tissue on a tissue microarray. Gain- and loss-of-function assays were performed to explore the role of DPYSL2 in bladder cancer growth and metastasis *in vitro* and *in vivo*. A proteomics assay was conducted to identify the interacting partners of DPYSL2. Our results suggest that DPYSL2 binds to PKM2 and inhibits the formation of tetrameric PKM2, which in turn promotes EMT and aerobic glycolysis in bladder cancer cells. This is the first report regarding the enhancive role of the DPYSL2/PKM2 axis in bladder cancer progression, providing the DPYSL2/PKM2 axis as a promising therapeutic target in bladder cancer treatment.

## Materials and Methods

### Patients and Tissue Samples

The fresh-frozen bladder tumor samples and the matched adjacent non-cancerous bladder tissue samples were collected from patients with primary bladder cancer before anticancer treatment from March 2018 to April 2019 at the Cancer Center of Guangzhou Medical University (Guangdong, China). Each patient provided written informed consent before sample collection. This study was approved by the Internal Review and Ethics Boards at the Cancer Center of Guangzhou Medical University.

A tissue microarray containing 176 bladder cancer tissue samples and 16 adjacent non-cancerous bladder tissue samples was purchased from Alenabio (#BL2081c; Xi’an, China). To compare the mRNA levels of DPYSL2 between patients with NMIBC and MIBC, the gene expression profiles (mRNA, normalized RNAseq FPKM-UQ) and clinicopathological information of 408 bladder cancer patients were obtained from the TCGA database (provisional) using cBioPortal^1^. The GSE89 and GSE32548 datasets were downloaded from the Gene Expression Omnibus (GEO) database. Other patient data were acquired from a cancer microarray database Oncomine.

### Animals

The animal experiments were approved by the experimental animal Ethics Committee of the Third Affiliated Hospital of Guangzhou Medical University and were conducted following the national statutory requirements and guidelines for the care and maintenance of experimental animals.

For tumor growth assay, female BALB/c nude mice (4-week old) were purchased from Guangdong Medical Laboratory Animal Center (Guangzhou, China). Human bladder cancer 5637 cells were stably transfected with empty lentiviral vectors (LV-control) or DPYSL2-overexpressing lentiviral vectors (LV-DPYSL2). Mice were randomly divided into two groups (*n* = 5/group) and were subcutaneously inoculated with LV-control- or LV-DPYSL2-transfected 5637 cells (4 × 10^6^) at the left and right armpits. Mice were euthanized at 4 weeks after inoculation, and the tumors were collected and weighed.

For tumor metastasis assay, female NOD-SCID mice (3-week old) were purchased from Charles River Laboratories in China (Beijing, China). Mice were randomly divided into two groups (*n* = 6/group) and received 2 × 10^6^ LV-control- or LV-DPYSL2-transfected 5637 cells via caudal vein injection. At 3 months after cancer cell injection, each mouse was injected with 0.15 mg/g potassium d-fluorescein through the tail vein. The luminescence was detected using an IVIS 200 imaging system (Xenogen, United States). Mice were then euthanized. The lungs were immediately collected, and the metastatic nodules were counted. Hematoxylin and eosin staining was performed to visualize the metastasis in the lung.

### Cell Lines and Cell Culture

HEK-293T and human bladder cancer cell lines 5637 and T24 were obtained from the American Type Culture Collection (Manassas, VA, United States) and cultured at 37°C in a humidified atmosphere with 5% CO_2_.

### Construction of Stable Cell Lines Overexpressing DPYSL2

T24 or 5637 cells were transfected with lentiviral vectors (pCDH, psPAX2, and pMD2 vectors) expressing Flag or DPYSL2-Flag, as previously described (Zou et al., 2019). The cell lines stably expressing DPYSL2 were transfected with lentiviral Plv5-GFP-Luc (GenePharma) for 5 days. The cell lines stably expressing Luc-DPYSL2-Flag were selected using 8 μg/mL puromycin for 2 weeks.

### RNA Interference

Third to sixth passages BCa cells are performed for RNA interference. The sequences of small interfering RNAs against DPYSL2 (siDPYSL2; GenePharma, Suzhou, China) were as follows: siDPYSL2 #1, 5′-GCCAGAUUUAUGAAGUACUTT-3′ (sense) and 5′-AGUACUUCAUAAAUCUGGCTT-3′ (anti-sense); siDPYSL2 #2, 5′-GCCCAUUGCACGUUUAACATT-3′ (sense) and 5′-UGU UAAACGUGCAAUGGGCTT-3′ (anti-sense). The sequences of siPKM2 (GenePharma) were 5′-GCCAUCUACCACUUGCAAUTT-3′ (sense) and 5′-AUUGC AAGUGGUAGAUGGCTT-3′ (anti-sense). The sequences of negative control siRNA (NC) were 5′-GCACAAGCUGGAG UACAACUACATT-3′ (sense) and 5′-UGUAGUUGUACUCCA GCUUGUGCTT-3′ (anti-sense). The siRNAs were transfected into 5637 or T24 cells using RNAiMAX (Invitrogen, Carlsbad, CA, United States) according to the manufacturer’s protocols.

### Cell Proliferation and Colony Formation Assays

T24 or 5637 cells were transfected with the corresponding siRNAs or plasmids and incubated for 24 h. For cell proliferation assay, cells were plated in 96-well culture plates at a density of 1 × 10^4^ cells/well and were counted at 24, 48, 72, 96, and 120 h after transfection. In the colony formation assay, cells were plated in 6-well plates at a density of 600 cells/well and cultured for 2 weeks. The cells were fixed with methanol and then stained with crystal violet, followed by colony counting. These experiments were repeated three times.

### Cell Migration and Invasion Assays

For cell migration assay, 1 × 10^5^ bladder cancer cells in 100 μL serum-free medium were added in the upper Transwell chamber (8.0-μm pore size; BD, San Jose, CA, United States). The upper chamber was coated with Matrigel for the invasion assay. Medium containing 10% fetal bovine serum was added to the bottom chambers. 5637 cells were allowed to migrate or invade for 24 or 72 h, respectively. T24 cells were allowed to migrate or invade for 12 or 48 h, respectively. The migrating or invading cells at the lower surface were fixed with methanol and stained with 1% crystal violet. Cells were counted, and images were acquired using a Nikon TI-S microscope (Nikon, Japan) at magnification 10×.

### Immunofluorescence Staining

Bladder cancer cells were fixed with 4% paraformaldehyde for 20 min and permeabilized with 0.1% Triton X-100 for 7 min, followed by incubation with anti-Flag (1:1000; ab1162; Abcam) or anti-DPYSL2 (1:1000; 10,188-1-AP; Proteintech) overnight at 4°C. Cells were then incubated with the corresponding Alexa Fluor 488–conjugated secondary IgG for 30 min. The nuclei were stained with DAPI. The results were examined using a confocal laser-scanning microscope (Nikon, Japan).

### Immunohistochemical (IHC) Staining

Paraffin-embedded tissue microarray was deparaffinized in xylene and ethanol (100%, 90%, and 75%, respectively) and rehydrated. The microarray was boiled in 5,000 mL EDTA (pH = 8.0) for 5 min at high pressure, and endogenous peroxidase was quenched using 0.5% hydrogen peroxide. After washing three times with PBS, the microarray was blocked with 7% normal goat serum and incubated with anti-DPYSL2 antibody (1:100) overnight at 4°C. The results were visualized using a Dako Chem-Mate EnVision kit (K500711; Dako, Agilent Technologies, Santa Clara, CA, United States) and were assessed by two independent pathologists in a blinded manner. The intensity of the staining was scored as previously described (Zou et al., 2019).

### Quantitative Real-Time PCR (qRT-PCR)

The total RNA in 5637 or T24 cells was isolated using TRIzol (Invitrogen). cDNA was synthesized using a PrimeScript^TM^ reverse transcription system (Takara, Japan). QRT-PCR was performed using an SYBR Premix Ex Taq^TM^ II kit (TaKaRa) and primers as follows: DPYSL2, forward: 5′-CCTTCCTCGTGTACATGGCTT-3′, reverse: 5′-ATTTTCTGCGTGGACTTGGGCTA-3′; GAPDH, forward: 5′-CCCACTCCTCCACCTTTGAC-3′, reverse: 5′-TCT TCCTCTTGTGCTCTTGC-3′.

### Western Blot Analysis

The cells or ground tissue samples were lysed in 1 × sodium dodecyl sulfate-polyacrylamide gel electrophoresis (SDS-PAGE) buffer. The protein concentrations were determined using a bicinchoninic acid kit (A53226; Thermo Fisher Scientific, Waltham, MA, United States). The protein samples were separated on a 10% SDS-PAGE gel and then transferred to a polyvinylidene fluoride membrane. After incubated with 5% bovine serum albumin at room temperature for 2 h, The membrane was incubated with primary antibody against DPYSL2 (1:1000; 10,188-1-AP; Proteintech), Flag (1:1000; ab1162; Abcam, Cambridge, United Kingdom), PKM1 (1:2000; 15,821-1-AP; Proteintech), PKM2 (1:2000; 15,822-1-AP; Proteintech), E-cadherin (1:2000; #14,472; CST, Danvers, MA, United States), Vimentin (1:2000; #5741; CST), ZEB (1:1000; #9585; CST), or β-actin (1:4000; sc-81178; Santa Cruz Biotechnology, Dallas, TX, United States) overnight at 4°C. After incubation with a horseradish peroxidase-conjugated secondary antibody for 1.5 h at room temperature, the protein bands were detected using a ChemiDOC XRS System (Bio-Rad, Hercules, CA, United States).

### Co-immunoprecipitation (Co-IP) and Silver Staining Assays

Cells at 80–90% confluency were transfected with corresponding plasmids and cultured for 48 h. Cells were lysed in IP lysis buffer (P0013; Beyotime, Shanghai, China) for 30 min at 4°C and then centrifuged at 12,000 × *g* for 10 min at 4°C. Equal amounts of cell lysates were incubated with anti-Flag antibody-conjugated protein A/G agarose beads (Santa Cruz) to obtain the immune complexes. After separating the complexes, the proteins were detected using a silver staining kit following the manufacturer’s protocol (P0017S; Beyotime). The corresponding band was cut from the gel and digested using in-gel trypsin. The protein mixture was analyzed using a nano-LC-MS/MS system (TripleTOF 5600; AB SCIEX, Concord, ON, United States). The mass spectrometry proteomics data for identification of proteins that interacted with DPYSL2 have been deposited to the ProteomeXchange Consortium^2^ via the iProX (PXD023279).

### Cross-Linking Analysis

The cells were lysed at 4°C for 30 min using 0.5% Triton X-100 sodium phosphate buffer (pH 7.3). After centrifugation at 2,000 rpm for 10 min, the supernatant of the cell lysate was immediately treated with 1% formaldehyde for 30 min. The reaction was stopped using 50 mM Tris–HCl (pH 8.0). The expression of PKM2 monomer, dimer, and tetramer were determined using Western blot analysis.

### Measurement of Glucose Uptake

Bladder cancer cells were transfected with the corresponding siRNAs or plasmids and cultured for 36 h, followed by incubation with RPMI 1640 medium without L-glucose and phenol red for an additional 8 h. The amount of glucose in the medium was determined using a glucose colorimetric assay kit (K606-100; BioVision, Milpitas, CA, United States), following the manufacturer’s protocols.

### Measurement of Lactate Production

Bladder cancer cells were transfected with the corresponding plasmids or siRNAs and cultured for 48 h. The lactate concentration in the culture medium was determined using a lactate colorimetric assay kit II (K627-100; BioVision), following the manufacturer’s instructions.

### Statistical Analysis

Data were presented as the means ± standard error of the mean. Statistical analysis was performed using the Prism 5 software (GraphPad, San Diego, CA, United States). The two groups were compared using the two-tailed unpaired Student *t*-test. The survival curves were generated using the Kaplan–Meier method and were compared using the log-rank test. A *P-*value < 0.05 was considered statistically significant.

## Results

### DPYSL2 Upregulation Correlates With Tumor Staging and Poor Prognosis in Bladder Cancer

**FIGURE 1:**
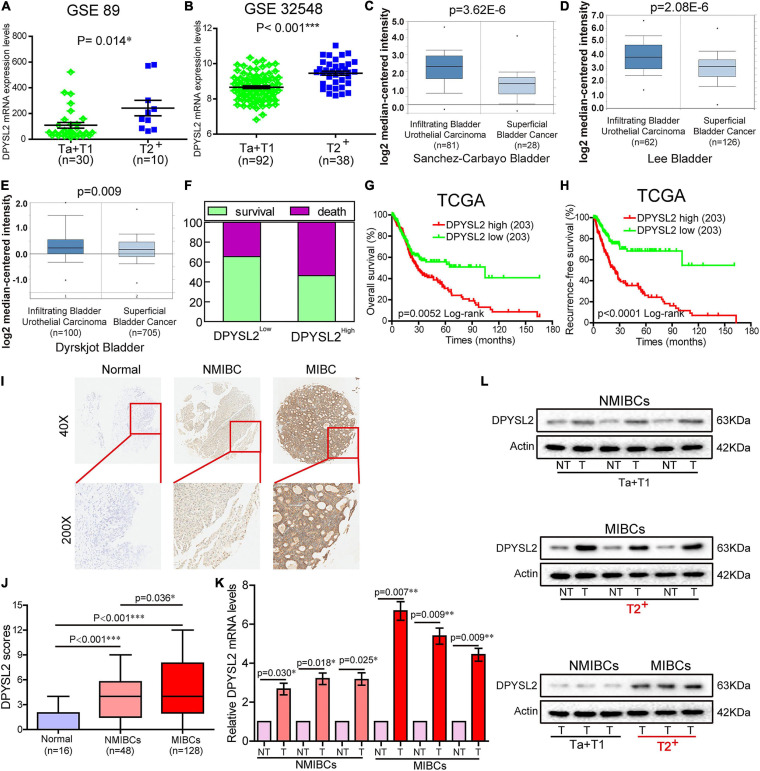
Dihydropyrimidinase like 2 (DPYSL2) upregulation correlated with tumor staging and poor survival in patients with bladder cancer. **(A,B)** In the GSE89 and GSE32548 datasets, DPYSL2 mRNA levels were significantly higher in muscular invasive bladder cancer (MIBC) samples (≥T2) than those in non-muscular invasive bladder cancer (NMIBC) samples (Ta and T1). **(C–E)** DPYSL2 mRNA levels were significantly increased in infiltrating bladder urothelial carcinoma compared with those in superficial bladder cancer in the Oncomine database. **(F–H)** In a TCGA bladder cancer specimen cohort (*n* = 406), the patients with high DPYSL2 expression had increased death rates **(F)** and decreased overall survival **(G)** and recurrence-free survival **(H)**, compared with the patients with low DPYSL2 expression. **(I,J)** Immunohistochemical staining was performed to detect the protein levels of DPYSL2 in a bladder cancer tissue microarray containing non-cancerous (*n* = 16), NMIBC (*n* = 48), and MIBC (*n* = 128) tissue samples. Representative images are shown. Magnification 40×, 200×. **(K,L)** Quantitative real-time PCR (qRT-PCR) and Western blot analysis were conducted to measure mRNA and protein levels of DPYSL2 in randomly selected paired fresh-frozen bladder cancer and adjacent non-cancerous bladder tissue samples. Actin was used as an internal reference. Data are expressed as the mean ± standard error of the mean (SEM). *P < 0.05, **P < 0.01, ***P < 0.001; *n* = 6. T, tumor; TCGA, The Cancer Genome Atlas; DPYSL2, dihydropyrimidinase like 2; NMIBC, non-muscular invasive bladder cancer; MIBC, muscular invasive bladder cancer; NT, non-cancerous tissue.

According to the degree of tumor invasion into the bladder wall, bladder cancer can be divided into NMIBC (stage Tis, Ta, and T1) and more aggressive MIBC (stages T2–T4) (Moch et al., 2016). To explore the association of DPYSL2 expression with bladder cancer staging and prognosis, we downloaded corresponding information from multiple databases. By analyzing the GSE89 and GSE32548 datasets from the GEO database, we found that DPYSL2 mRNA levels were significantly increased in the bladder tumor tissue samples from patients with MIBC compared with those from patients with NMIBC (Figures 1A,B). Similar results were observed in the samples obtained from the Oncomine database (Figures 1C–E). Consistently, after dividing the TCGA dataset into low and high DPYSL2 expression groups according to the median value of DPYSL2 mRNA levels, we found that patients with high DPYSL2 mRNA levels exhibited increased mortality compared with those with low DPYSL2 mRNA levels (Figure 1F). High DPYSL2 expression significantly correlated with decreased overall survival (Figure 1G) and recurrence-free survival (Figure 1H) of patients with bladder cancer. In addition, DPYSL2 expression positively correlated with T stage (*P* = 0.015), N stage (*P* = 0.011), recurrence and progression (*P* = 0.012), as well as clinical stage (*P* < 0.001) (Table 1) in bladder cancer patients.

**TABLE 1:** Comparison of clinical features between bladder cancer patients with low and high^#^ DPYSL2 mRNA levels in the TCGA database.

Clinical character	Clinical groups	DPYSL2		*x*^2^, df	*p-*value
				
		High (*n* = 203) (%)	Low (*n* = 203) (%)		
Age (years)	≤60	49 (24.1)	58 (28.6)	1.028, 1	0.311
	>60	154 (75.9)	145 (71.4)		
Gender	Male	144 (70.9)	155 (76.4)	1.536, 1	0.215
	Female	59 (29.1)	48 (23.6)		
Histological subtype***	Papillary	45 (22.2)	86 (42.4)	18.76, 1	<0.001
	Non-papillary	155 (76.4)	115 (56.7)		
pTstatus*	T1	2 (1.0)	2 (1.0)	10.50, 3	0.015
	T2	48 (23.6)	70 (34.5)		
	T3	108 (53.8)	85 (41.9)		
	T4	37 (18.2)	21 (10.3)		
pN status*	N0	109 (53.7)	127 (62.6)	6.481, 1	0.011
	N1∼ N2	77 (37.9)	51 (25.1)		
Recurred/Progressed*	No	73 (36.0)	104 (51.2)	5.627, 1	0.012
	Yes	77 (37.9)	64 (31.5)		
Clinical Stage***	Stage I∼II	48 (23.6)	83 (40.9)	14.36, 1	<0.001
	Stage III∼IV	155 (76.4)	118 (58.1)		

T, tumor size; N, lymph node status; DPYSL2, Dihydropyrimidinase like 2; TCGA, The Cancer Genome Atlas; ^#^, TCGA dataset was divided into low and high DPYSL2 expression groups according to the median value of DPYSL2 mRNA levels.

*P < 0.05, ***P < 0.001.

Then, we sought to verify the expression pattern of DPYSL2 in different stages of bladder cancer at the protein level. IHC staining revealed that bladder cancer tissue had remarkably increased DPYSL2 protein expression compared with normal tissue and that MIBC tissue exhibited considerably higher DPYSL2 protein levels than NMIBC tissue (Figures 1I,J). qRT-PCR and Western blot analysis consistently showed that the mRNA and protein levels of DPYSL2 were significantly elevated in fresh-frozen bladder cancer tissue samples compared with those in adjacent non-cancerous samples, as well as in MIBC samples compared with those in NMIBC samples (Figures 1K,L). Together, these results suggest that DPYSL2 upregulation is associated with tumor staging and poor prognosis in bladder cancer patients.

### DPYSL2 Promotes Bladder Cancer Progression *in vitro* and *in vivo*

**FIGURE 2:**
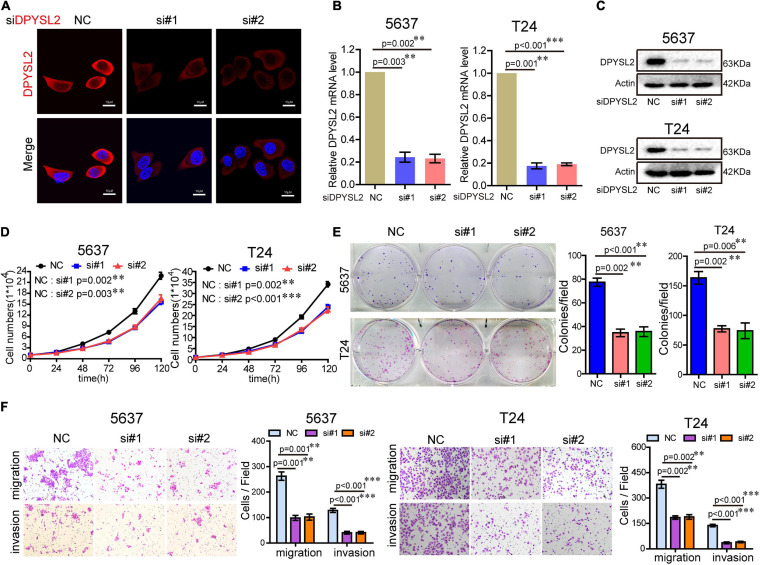
Knockdown of DPYSL2 inhibited cell proliferation, colony formation, migration, and invasion of bladder cancer cells. **(A)** T24 cells were transfected with small interfering RNA against DPYSL2 (siDPYSL2) or negative control. DPYSL2 was immunostained with an anti-DPYSL2 antibody and detected by immunofluorescence microscopy. Representative images are shown. Magnification 200×. **(B,C)** 5637 and T24 cells were transfected with siDPYSL2 or negative control. qRT-PCR and Western blot analysis were performed to measure the mRNA and protein levels of DPYSL2. **(D–F)** Cell proliferation, colony formation, migration, and invasion assays were conducted. Data are expressed as the mean ± SEM. **P < 0.01, ***P < 0.001; *n* = 3. NC, negative control; si, small interfering RNA.

**FIGURE 3:**
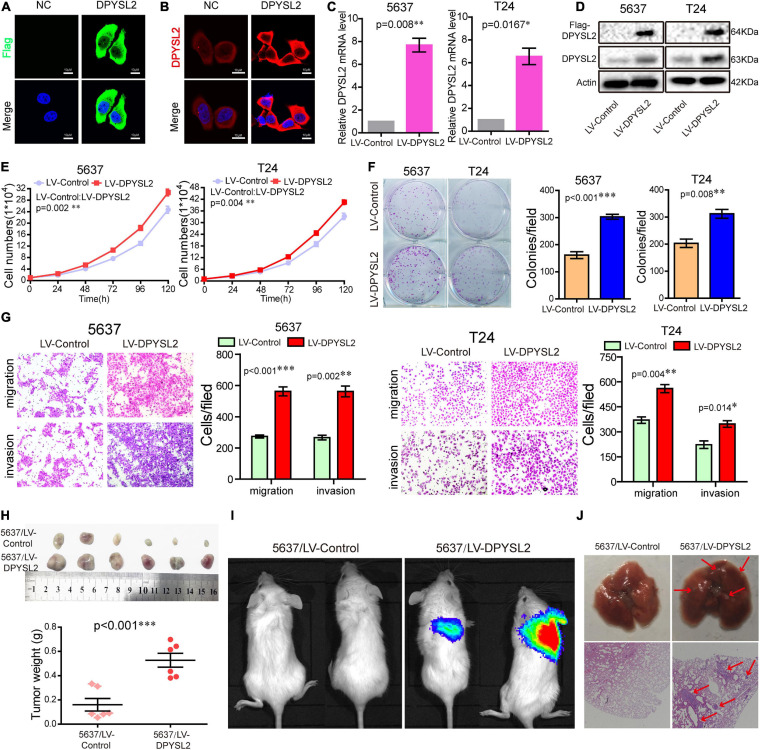
Dihydropyrimidinase like 2 (DPYSL2) overexpression promoted bladder cancer cell proliferation, colony formation, migration, and invasion *in vitro*, as well as tumor growth and metastasis *in vivo*. **(A,B)** T24 cells were transfected with the Flag-DPYSL2 plasmids or negative control. Flag and DPYSL2 were immunostained with anti-Flag **(A)** or anti-DPYSL2 **(B)** antibody. Representative images are shown. Magnification 200×. **(C,D)** 5637 and T24 cells were stably transfected with empty lentiviral vectors (LV-control) or lentiviral vectors overexpressing DPYSL2 (LV-DPYSL2). qRT-PCR and Western blot analysis were conducted to measure the mRNA and protein levels of DPYSL2. **(E–G)** Cell proliferation, colony formation, migration, and invasion assays were conducted. **(H)** NOD-SCID mice were subcutaneously inoculated with 4 × 10^6^ LV-control- or LV-DPYSL2-transfected 5637 cells at the left and right armpits (*n* = 5/group). Mice were euthanized at 4 weeks after inoculation, and the tumors were collected, measured, and weighed. Data are expressed as the mean ± SEM. *P < 0.05, **P < 0.01, ***P < 0.001; *n* = 5. **(I)** NOD-SCID mice were injected with 2 × 10^6^ LV-control- or LV-DPYSL2-transfected 5637 cells through caudal vein (*n* = 6/group). At 3 months after injection, each mouse was injected with 0.15 mg/g potassium d-fluorescein through the tail vein. The IVIS 200 imaging system was used to detect luminescence in tumor-bearing mice. **(J)** Upper panel: Representative images of pulmonary metastases. Lower panel: Hematoxylin and eosin staining of pulmonary metastases. Red arrows indicate metastases.

To explore the role of DPYSL2 in bladder cancer progression, we performed gain- and loss-of-function assays in 5637 and T24 cell lines. 5637 is a human superficial bladder cancer cell line, while T24 is a human bladder transitional cell cancer cell line. The basal level of DPYSL2 in 5637 and T24 cells were detected. The results show that the basal level of DPYSL2 in T24 cells is obviously higher than that in 5637 cells (Supplementary Figure 1). Figures 2A–C shows that DPYSL2 was mainly localized in the cytoplasm of T24 cells and that siDPYSL2 transfection dramatically silenced DPYSL2 mRNA and protein expression in both 5637 and T24 cell lines. Compared with negative control, siDPYSL2-mediated DPYSL2 silencing significantly inhibited cell proliferation (Figure 2D), colony formation (Figure 2E), migration, and invasion (Figure 2F) of 5637 and T24 cells. On the other hand, Figures 3A–D show that transfection with DPYSL2-overexpressing vectors resulted in dramatic overexpression of DPYSL2 in both cell lines. In contrast to DPYSL2 silencing, DPYSL2 overexpression significantly promoted cell proliferation (Figure 3E), colony formation (Figure 3F), migration, and invasion (Figure 3G) in both cell lines. These results suggest that DPYSL2 is required for bladder cancer development.

We further explored the role of DPYSL2 in bladder cancer progression *in vivo*. We found that the nude mice transplanted with DPYSL2-overexpressing 5637 cells had significantly greater tumor mass (Figure 3H) and more metastatic nodules in the lungs (Figures 3I,J) than those transplanted with control cells. Collectively, these data suggest that DPYSL2 promotes bladder cancer progression and metastasis *in vitro* and *in vivo*.

### DPYSL2 Binds to PKM2 and Hinders the Formation of PKM2 Tetramer

**FIGURE 4:**
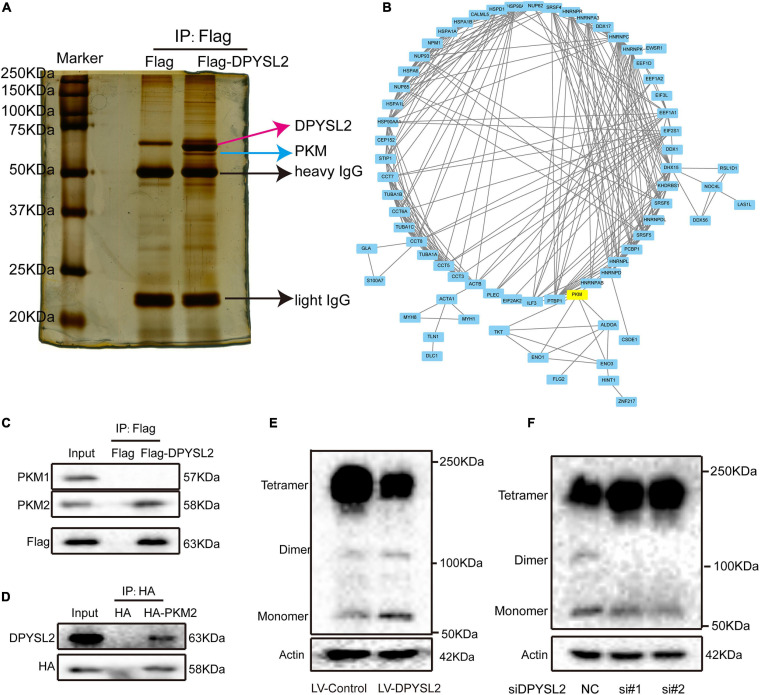
Dihydropyrimidinase like 2 (DPYSL2) interacted with pyruvate kinase M2 (PKM2) in bladder cancer cells. **(A)** Co-immunoprecipitation (Co-IP), followed by silver staining and mass spectrometry, was performed to identify the proteins that physically interacted with DPYSL2. **(B)** A protein-protein interaction network was established to predict the interactions of DPYSL2 with potential candidate proteins using the STRING database (https://string-db.org/). **(C)** HEK293T cells were transfected with Flag or Flag-DPYSL2 plasmids. The Flag-DPYSL2 complexes were co-immunoprecipitated using an anti-Flag antibody, followed by Western blot analysis to detect PKM1 and PKM2. **(D)** HEK293T cells were transfected with HA or HA-PKM2 plasmids. The HA-PKM2 complexes were co-immunoprecipitated using an anti-HA antibody, followed by Western blot analysis to detect DPYSL2. **(E,F)** T24 cells were transfected with LV-DPYSL2, siDPYSL2, or corresponding negative control. A cross-link analysis was performed to detect the monomers, dimers, and tetramers of PKM2.

To investigate the molecular mechanisms underlying the enhancive role of DPYSL2 in bladder cancer progression, we performed a proteomics assay to identify the proteins that physically interact with DPYSL2. A Co-IP assay followed by silver staining showed that a band of about 63 kDa was present in DPYSL2-overexpressing cell lysates, but not in the control cell lysates (Figure 4A). Mass spectrometry analysis revealed that a total of 119 proteins physically interacted with DPYSL2 (Supplementary Table 1). A protein-protein interaction network of these proteins was established using the STRING database^3^ (Figure 4B). Among these proteins, we selected cancer progression-related PKM2 for further investigation (Zhang et al., 2019). The DPYSL2 (Supplementary Figure 2A) protein and PKM protein (Supplementary Figure 2B) were identified by mass spectrometry. Co-IP further confirmed that DPYSL2 binds to PKM2, but not PKM1 (Figures 4C,D).

Next, we explored whether DPYSL2 affects the protein expression of PKM2. Western blot analysis showed that neither DPYSL2 overexpression (Supplementary Figure 3A) nor DPYSL2 silencing (Supplementary Figure 3B) affected PKM2 protein expression. However, the cross-linking analysis revealed that DPYSL2 overexpression significantly suppressed the formation of PKM2 tetramers while enhancing the formation of PKM2 monomers and dimers (Figure 4E). DPYSL2 silencing showed contrasting results (Figure 4F). These data suggest that DPYSL2 binds to PKM2 and deactivates PKM2 by hindering the formation of PKM2 tetramers.

### DPYSL2 Promotes Bladder Cancer Cell Malignant Phenotypes Through PKM2

**FIGURE 5:**
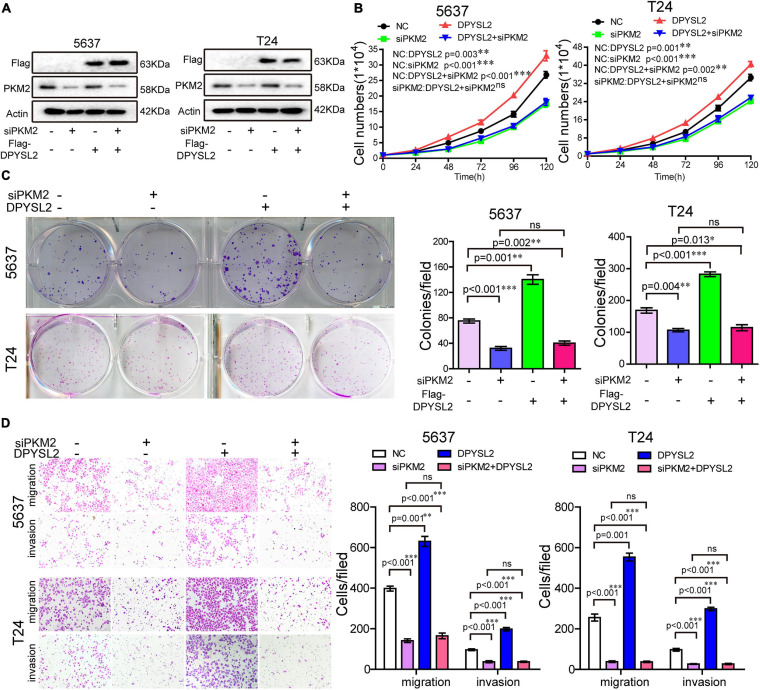
Pyruvate kinase M2 (PKM2) silencing completely abolished DPYSL2-enhanced malignant behaviors of bladder cancer cells. Stable Flag-DPYSL2-overexpressing 5637 or T24 cells were transfected with siPKM2 or negative control. The protein levels of Flag-DPYSL2 and PKM2 **(A)**, cell proliferation **(B)**, colony formation **(C)**, migration, and invasion **(D)** were examined. Data are expressed as the mean ± SEM. *P < 0.05, **P < 0.01, ***P < 0.001; ns, non-significant; *n* = 3.

Next, we sought to investigate whether PKM2 mediates the enhancive role of DPYSL2 in the malignant behavior of bladder cancer cells by silencing PKM2 expression in DPYSL2-overexpressing bladder cancer cells. Figure 5A shows that siPKM2 transfection effectively silenced PKM2 protein expression, even when DPYSL2 was overexpressed. Compared with negative control, PKM2 silencing not only substantially inhibited bladder cancer cell proliferation, clone formation, migration, and invasion but also completely abolished DPYSL2-induced enhancement of these malignant behaviors (Figures 5B–D). These findings suggest that DPYSL2 promotes bladder cancer progression through PKM2.

### DPYSL2 Promoted Aerobic Glycolysis and EMT Through PKM2

**FIGURE 6:**
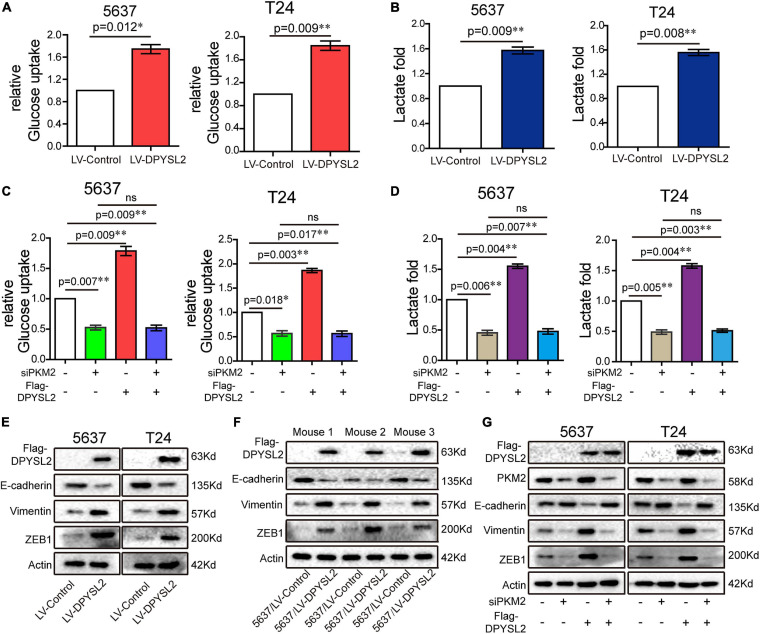
Dihydropyrimidinase like 2 (DPYSL2) promoted aerobic glycolysis and epithelial–mesenchymal transition (EMT) via PKM2. **(A,B)** Glucose uptake **(A)** and lactate production **(B)** were measured in 5637 and T24 cell lines stably overexpressing DPYSL2. **(C,D)** 5637 or T24 cells were transfected with Flag-DPYSL2 plasmids and siPKM2 individually or in combination. Glucose uptake **(C)** and lactate production **(D)** were measured, respectively, at 36 or 48 h after transfection. **(E,F)** Western blot analysis was performed to determine EMT marker expression in 5637 and T24 cell lines stably overexpressing DPYSL2 **(E)** and in three pairs of mouse tumors **(F)**. **(G)** 5637 or T24 cells Flag-DPYSL2 plasmids and siPKM2 individually or in combination. Western blot analysis was performed to measure EMT marker expression. Actin was used as an internal reference. Data are expressed as the mean ± SEM. *P < 0.05, **P < 0.01; ns, non-significant; *n* = 3.

Next, we further explored the role of DPYSL2/PKM2 in aerobic glycolysis and EMT of bladder cancer cells. We found that DPYSL2 overexpression promoted glucose uptake (Figure 6A) and lactate production (Figure 6B), whereas DPYSL2 silencing inhibited glucose uptake (Supplementary Figure 4A) and lactate production (Supplementary Figure 4B) in bladder cancer cells. PKM2 silencing completely abolished DPYSL2-enhanced glucose uptake (Figure 6C) and lactate production (Figure 6D) in bladder cancer cells.

**FIGURE 7:**
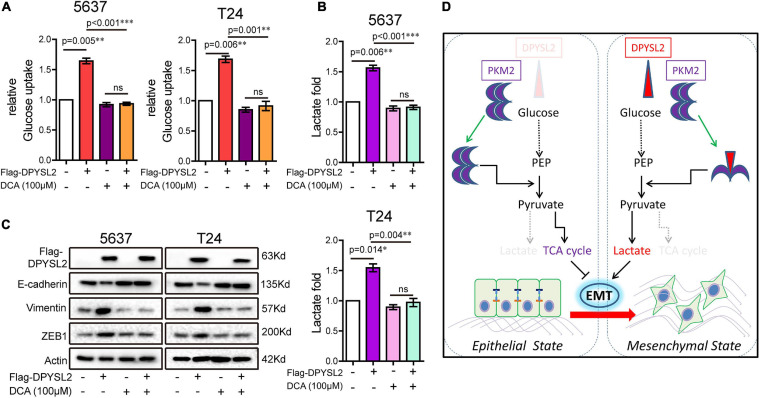
Glycolysis inhibitor dichloroacetate (DCA) abolished DPYSL2-induced aerobic glycolysis and EMT marker alterations in bladder cancer cells. **(A–C)** 5637 or T24 cells were transfected with Flag-DPYSL2 and incubated for 12 h, followed by DCA (100 μM) treatment for 36 h. Glucose uptake **(A)**, lactate production **(B)**, and protein levels of EMT markers **(C)** were measured. **(D)** A schematic diagram illustrates that DPYSL2 promotes EMT and aerobic glycolysis by interacting with PKM2. *P < 0.05, **P < 0.01, ***P < 0.001; ns, non-significant.

Considering that PKM2 promotes EMT in cancer cells (Hamabe et al., 2014), we examined whether DPYSL2 affects EMT in bladder cancer cells. As shown in Figure 6E, DPYSL2 overexpression significantly attenuated E-cadherin protein expression while enhancing Vimentin and ZEB1 protein expression in bladder cancer cells. Contrasting results were observed in DPYSL2-silenced cells (Supplementary Figure 4C). Consistent results were observed *in vivo* (Figure 6F). Importantly, PKM2 silencing completely blocked DPYSL2-induced EMT marker alterations in bladder cancer cells (Figure 6G), suggesting that DPYSL2 promotes EMT through PKM2. Then, we examined the effect of DPYSL2 overexpression on aerobic glycolysis and EMT in the presence of glycolysis inhibitor dichloroacetate (DCA). We found that DPYSL2 failed to promote glucose uptake (Figure 7A), lactate production (Figure 7B), and EMT (Figure 7C) in bladder cancer cells in the presence of DCA, suggesting that active glycolysis is required for DPYSL2-induced EMT in bladder cancer.

## Discussion

In this study, we demonstrated that the upregulation of DPYSL2 expression correlated with cancer staging and poor prognosis in patients with bladder cancer. DPYSL2 promoted bladder cancer cell proliferation, migration, invasion, and colony formation *in vitro*, as well as xenograft tumor growth and lung metastasis *in vivo*. Mechanistically, DPYSL2 bound to PKM2 and blocked the formation of highly catalytic PKM2 tetramer, which in turn promotes anaerobic glycolysis and EMT in bladder cancer cells.

Previous studies have shown that DPYSL2 is differentially expressed in various tumors, with increased expression in non-small cell lung cancer (NSCLC) (Oliemuller et al., 2013) and colorectal carcinoma (Wu et al., 2008) and decreased expression in breast cancer (Shimada et al., 2014). The expression pattern of DPYSL2 in bladder cancer has not been reported yet. In this study, we, for the first time, demonstrated that bladder cancer tissue had remarkably increased DPYSL2 protein expression compared with adjacent non-cancerous tissue on a tissue microarray. Furthermore, by analyzing patient data from tissue microarray and different databases, we found that MIBC tissue exhibited considerably higher DPYSL2 mRNA and protein levels than NMIBC tissue. It has been reported that increased levels of nuclear phosphorylated DPYSL2 correlate with poor prognosis in patients with NSCLC (Oliemuller et al., 2013), but has no association with clinical outcome, tumor size, lymph node metastasis, and histological grade in patients with breast cancer (Shimada et al., 2014). In this study, we demonstrated that in the TCGA dataset, high DPYSL2 expression in bladder cancer tissue significantly correlated with decreased overall survival and recurrence-free survival, along with increased T stage, N stage, clinical stage, and tumor recurrence and progression in patients. These results collectively suggest that DPYSL2 upregulation is closely associated with tumorigenesis and tumor progression in bladder cancer and may present as a diagnostic and prognostic biomarker for bladder cancer.

To explore the function of DPYSL2 in bladder cancer progression, we performed gain- and loss-of-function assays. Immunofluorescent staining showed that DPYSL2 protein was mainly expressed in the cytoplasm of 5637 bladder cancer cells, consistent with the findings of Wu et al. (2008) that DPYSL2 is predominantly located in the cytoplasm of colorectal carcinoma cell cells. We also observed that DPYSL2 promoted cell proliferation, colony formation, migration, and invasion *in vitro*, as well as tumor formation and lung metastasis *in vivo*. Consistently, Moutal et al. (2018) have demonstrated that DPYSL2 expression and phosphorylation drive glioblastoma proliferation and survival *in vitro* and *in vivo*. These findings suggest that DPYSL2 may participate in oncogenic and metastatic mechanisms in bladder cancer.

To further investigate the mechanism underlying the functions of DPYSL2 in bladder cancer, we performed proteomic analysis to identify the interacting partners of DPYSL2. Based on the results of Co-IP and LC-MS/MS analysis, we selected cancer progression-related PKM2 as the candidate protein. Co-IP and cross-link analysis showed that DPYSL2 physically bound to PKM2 and inhibited the formation of PKM2 tetramers while promoting the formation of PKM2 monomers and dimers. It is well-established that the less active PKM2 dimer drives aerobic glycolysis, whereas the active PKM2 tetramer produces pyruvate for oxidative phosphorylation (Christofk et al., 2008; Hitosugi et al., 2009; Anastasiou et al., 2012). The mechanisms involved in the PKM2 dimer-tetramer dynamics include fructose-1,6-biphosphate-mediated PKM2 tetramerization (Dombrauckas et al., 2005), tyrosine phosphorylation (Yang, 2015), acetylation (Prakasam et al., 2018), and modulation by small molecule PKM2 activators (Wubben et al., 2020). In the present study, we identified DPYSL2 as a novel regulator that promotes the conversion of PKM2 tetramers to dimers.

Recently, several new oncogenic drivers have been shown to play pivotal roles in EMT during tumor progression (Xu et al., 2015; Li et al., 2018; Zeng et al., 2018). Studies have reported that PKM2 dimer can translocate to the nucleus, promoting EMT in colorectal cancer (Hamabe et al., 2014) and oral squamous cell carcinoma (Tanaka et al., 2018). Since PKM2 dimer-tetramer dynamics plays a key role in aerobic glycolysis and EMT in cancer (Zhang et al., 2019), we questioned whether DPYSL regulates aerobic glycolysis and EMT in bladder cancer. Indeed, DPYSL2 overexpression promoted glucose uptake and lactate production in bladder cancer cells. DPYSL2 overexpression also significantly attenuated E-cadherin protein expression, while enhancing Vimentin and ZEB1 protein expression in bladder cancer cells. Importantly, these effects were completely blocked by PKM2 silencing, suggesting that DPYSL2 promotes aerobic glycolysis and EMT via PKM2 in bladder cancer. In addition, DPYSL2 failed to promote glucose uptake, lactate production, and EMT in bladder cancer cells in the presence of glycolysis inhibitor DCA, suggesting that active glycolysis is required for DPYSL2-induced EMT in bladder cancer.

## Conclusion

In this study, we demonstrated that DPYSL2 upregulation correlated with tumor staging and poor prognosis in patients with bladder cancer. Functionally, DPYSL2 promoted the malignant behavior of bladder cancer cells *in vitro* and tumor growth and distant metastasis *in vivo*. Mechanistically, DPYSL2 bound to PKM2 and induced the conversion of PKM2 tetramers to PKM2 dimers, thus promoting aerobic glycolysis and EMT in bladder cancer cells (Figure 7D). These results suggest that DPYSL2 plays an oncogenic role in bladder cancer through allosteric modulation of PKM2.

## Data Availability Statement

The datasets presented in this study can be found in online repositories. The names of the repository/repositories and accession number(s) can be found below: ProteomeXchange Consortium (http://proteomecentral.proteomexchange.org) via the iProX (PXD023279).

## Ethics Statement

The animal experiments were approved by the experimental animal Ethics Committee of the Third Affiliated Hospital of Guangzhou Medical University and were conducted following the National Statutory requirements and guidelines for the care and maintenance of experimental animals. Each patient provided written informed consent before sample collection. This study was approved by the Internal Review and Ethics Boards at the Cancer Center of Guangzhou Medical University.

## Author Contributions

JZ provided substantial contributions to the conception and design of the present study. YC, HL, and XH were responsible for the analysis and interpretation of the data. PL and SC performed Western blot analysis, qRT-PCR, and RNA interference experiments. JZ and RH performed the experiments. JZ and YC provided statistical support and analyzed the IHC data. All authors drafted the present manuscript and read and approved the final manuscript.

## Conflict of Interest

The authors declare that the research was conducted in the absence of any commercial or financial relationships that could be construed as a potential conflict of interest.

## Footnotes

1 http://www.cbioportal.org/

2 http://proteomecentral.proteomexchange.org

3 https://string-db.org/

## References

[B1] Aiello N. M. Kang Y. (2019). Context-dependent EMT programs in cancer metastasis. *J. Exp. Med.* 216 1016–1026. 10.1084/jem.20181827 30975895 PMC6504222

[B2] Anastasiou D. Yu Y. Israelsen W. J. Jiang J. K. Boxer M. B. Hong B. S. (2012). Pyruvate kinase M2 activators promote tetramer formation and suppress tumorigenesis. *Nat. Chem. Biol.* 8 839–847. 22922757 10.1038/nchembio.1060 PMC3711671

[B3] Antoni S. Ferlay J. Soerjomataram I. Znaor A. Jemal A. Bray F. (2017). Bladder cancer incidence and mortality: a global overview and recent trends. *Eur. Urol.* 71 96–108. 10.1016/j.eururo.2016.06.010 27370177

[B4] Bladder Source Globocan (2018). 2019.ABCDEFG. Chennai: https://gco. iarc.fr/today/data/factsheets/cancers/30-Bladder-fact-sheet.pdf.

[B5] Cai G. Wu D. Wang Z. Xu Z. Wong K. B. Ng C. F. (2017). Collapsin response mediator protein-1 (CRMP1) acts as an invasion and metastasis suppressor of prostate cancer via its suppression of epithelial-mesenchymal transition and remodeling of actin cytoskeleton organization. *Oncogene.* 36 546–558. 10.1038/onc.2016.227 27321179 PMC5290039

[B6] Chang Y. H. Tsai J. N. Chang S. W. Hsu W. T. Yang C. P. Hsiao C. W. (2020). Regulation of adipogenesis and lipid deposits by collapsin response mediator protein 2. *Int. J. Mol. Sci.* 21:2172. 10.3390/ijms21062172 32245267 PMC7139951

[B7] Chen M. Sheng X. J. Qin Y. Y. Zhu S. Wu Q. X. Jia L. (2019). TBC1D8 amplification drives tumorigenesis through metabolism reprogramming in ovarian cancer. *Theranostics.* 9 676–690. 10.7150/thno.30224 30809301 PMC6376479

[B8] Christofk H. R. Vander Heiden M. G. Wu N. Asara J. M. Cantley L. C. (2008). Pyruvate kinase M2 is a phosphotyrosine-binding protein. *Nature* 452 181–186. 10.1038/nature06667 18337815

[B9] Dombrauckas J. D. Santarsiero B. D. Mesecar A. D. (2005). Structural basis for tumor pyruvate kinase M2 allosteric regulation and catalysis. *Biochemistry* 44 9417–9429. 10.1021/bi0474923 15996096

[B10] Goulet A. C. Watts G. Lord J. L. Nelson M. A. (2007). Profiling of selenomethionine responsive genes in colon cancer by microarray analysis. *Cancer Biol. Ther.* 6 494–503. 10.4161/cbt.6.4.3813 17374985

[B11] Hamabe A. Konno M. Tanuma N. Shima H. Tsunekuni K. Kawamoto K. (2014). Role of pyruvate kinase M2 in transcriptional regulation leading to epithelial-mesenchymal transition. *Proc. Natl. Acad. Sci. U S A.* 111 15526–15531. 10.1073/pnas.1407717111 25313085 PMC4217454

[B12] Hitosugi T. Kang S. Vander Heiden M. G. Chung T. W. Elf S. Lythgoe K. (2009). Tyrosine phosphorylation inhibits PKM2 to promote the Warburg effect and tumor growth. *Sci. Signal.* 2:ra73. 10.1126/scisignal.2000431 19920251 PMC2812789

[B13] Islam S. S. Mokhtari R. B. Noman A. S. Uddin M. Rahman M. Z. Azadi M. A. (2016). Sonic hedgehog (Shh) signaling promotes tumorigenicity and stemness via activation of epithelial-to-mesenchymal transition (EMT) in bladder cancer. *Mol. Carcinog.* 55 537–551. 10.1002/mc.22300 25728352

[B14] Israelsen W. J. Vander Heiden M. G. (2015). Pyruvate kinase: function, regulation and role in cancer. *Semin. Cell. Dev. Biol.* 43 43–51. 10.1016/j.semcdb.2015.08.004 26277545 PMC4662905

[B15] Li M. Y. Liu J. Q. Chen D. P. Li Z. Y. Qi B. Yin W. J. (2018). p68 prompts the epithelial-mesenchymal transition in cervical cancer cells by transcriptionally activating the TGF-beta1 signaling pathway. *Oncol. Lett.* 15 2111–2116. 29434913 10.3892/ol.2017.7552 PMC5777103

[B16] Li Z. Yang P. Li Z. (2014). The multifaceted regulation and functions of PKM2 in tumor progression. *Biochim. Biophys. Acta.* 1846 285–296. 10.1016/j.bbcan.2014.07.008 25064846

[B17] Liao T. T. Yang M. H. (2017). Revisiting epithelial-mesenchymal transition in cancer metastasis: the connection between epithelial plasticity and stemness. *Mol. Oncol.* 11:792. 10.1002/1878-0261.12096 28649800 PMC5496497

[B18] Matsunuma R. Chan D. W. Kim B. J. Singh P. Han A. Saltzman A. B. (2018). DPYSL3 modulates mitosis, migration, and epithelial-to-mesenchymal transition in claudin-low breast cancer. *Proc. Natl. Acad. Sci. U S A.* 115 E11978–E11987. 30498031 10.1073/pnas.1810598115 PMC6305012

[B19] Mayumi T. Makoto S. Hideyuki S. (2012). Pyruvate kinase M2: multiple faces for conferring benefits on cancer cells. *Clin. Cancer Res.* 18 5554–5561. 10.1158/1078-0432.ccr-12-0859 23071357

[B20] Moch H. Cubilla A. L. Humphrey P. A. Reuter V. E. Ulbright T. M. (2016). The 2016 WHO classification of tumours of the urinary system and male genital organs-part a: renal, penile, and testicular tumours. *Eur. Urol.* 70 93–105. 10.1016/j.eururo.2016.02.029 26935559

[B21] Moutal A. Villa L. S. Yeon S. K. Householder K. T. Park K. D. Sirianni R. W. (2018). CRMP2 phosphorylation drives glioblastoma cell proliferation. *Mol. Neurobiol.* 55 4403–4416. 10.1007/s12035-017-0653-9 28660485 PMC5745298

[B22] Mrkvicova A. Chmelarova M. Peterova E. Havelek R. Baranova I. Kazimirova P. (2019). The effect of sodium butyrate and cisplatin on expression of EMT markers. *Plos One* 14:e0210889. 10.1371/journal.pone.0210889 30653577 PMC6336326

[B23] Nicolls M. R. D’Antonio J. M. Hutton J. C. Gill R. G. Czwornog J. L. Duncan M. W. (2003). Proteomics as a tool for discovery: proteins implicated in Alzheimer’s disease are highly expressed in normal pancreatic islets. *J. Proteome Res.* 2 199–205. 10.1021/pr025576x 12716134

[B24] Nieto M. A. Huang R. Y. Jackson R. A. Thiery J. P. (2016). Emt: 2016. *Cell* 166 21–45. 27368099 10.1016/j.cell.2016.06.028

[B25] Oliemuller E. Pelaez R. Garasa S. Pajares M. J. Agorreta J. Pio R. (2013). Phosphorylated tubulin adaptor protein CRMP-2 as prognostic marker and candidate therapeutic target for NSCLC. *Int. J. Cancer.* 132 1986–1995. 10.1002/ijc.27881 23023514

[B26] Prakasam G. Iqbal M. A. Bamezai R. N. K. Mazurek S. (2018). Posttranslational modifications of pyruvate kinase M2: tweaks that benefit cancer. *Front. Oncol.* 8:22. 10.3389/fonc.2018.00022 29468140 PMC5808394

[B27] Ritterson Lew C. Guin S. Theodorescu D. (2015). Targeting glycogen metabolism in bladder cancer. *Nat. Rev. Urol.* 12 383–391. 10.1038/nrurol.2015.111 26032551 PMC4678000

[B28] Rout-Pitt N. Farrow N. Parsons D. Donnelley M. (2018). Epithelial mesenchymal transition (EMT): a universal process in lung diseases with implications for cystic fibrosis pathophysiology. *Respir. Res.* 19:136. 10.1186/s12931-018-0834-8 PMC6052671 30021582

[B29] Shimada K. Ishikawa T. Nakamura F. Shimizu D. Chishima T. Ichikawa Y. (2014). Collapsin response mediator protein 2 is involved in regulating breast cancer progression. *Breast Cancer* 21 715–723. 10.1007/s12282-013-0447-5 23381229

[B30] Tanaka F. Yoshimoto S. Okamura K. Ikebe T. Hashimoto S. (2018). Nuclear PKM2 promotes the progression of oral squamous cell carcinoma by inducing EMT and post-translationally repressing TGIF2. *Oncotarget* 9 33745–33761. 10.18632/oncotarget.25850 30333907 PMC6173467

[B31] Tominaga K. Minato H. Murayama T. Sasahara A. Nishimura T. Kiyokawa E. (2019). Semaphorin signaling via MICAL3 induces symmetric cell division to expand breast cancer stem-like cells. *Proc. Natl. Acad. Sci. U S A.* 116 625–630. 10.1073/pnas.1806851116 30587593 PMC6329980

[B32] Warburg O. (1956). On the origin of cancer cells. *Science* 123 309–314. 13298683 10.1126/science.123.3191.309

[B33] Wu C. C. Chen H. C. Chen S. J. Liu H. P. Hsieh Y. Y. Yu C. J. (2008). Identification of collapsin response mediator protein-2 as a potential marker of colorectal carcinoma by comparative analysis of cancer cell secretomes. *Proteomics* 8 316–332. 10.1002/pmic.200700819 18203259

[B34] Wubben T. J. Pawar M. Weh E. Smith A. Sajjakulnukit P. Zhang L. (2020). Small molecule activation of metabolic enzyme pyruvate kinase muscle isozyme 2, PKM2, circumvents photoreceptor apoptosis. *Sci. Rep.* 10:2990. 10.1038/s41598-020-59999-w PMC7031539 32076076

[B35] Xu S. H. Huang J. Z. Xu M. L. Yu G. C. Yin X. F. Chen D. (2015). ACK1 promotes gastric cancer epithelial-mesenchymal transition and metastasis through AKT-POU2F1-ECD signalling. *J. Pathol.* 236 175–185. 10.1002/path.4515 25678401

[B36] Yang W. (2015). Structural basis of PKM2 regulation. *Protein Cell.* 6 238–240. 10.1007/s13238-015-0146-4 25773278 PMC4383752

[B37] Yutaka H. Yoshimitsu A. Yasuhito Y. (2013). Multiple-to-multiple relationships between microRNAs and target genes in gastric cancer. *Plos One.* 8:e62589. 10.1371/journal.pone.0062589 23667495 PMC3648557

[B38] Zeng B. Lin Z. W. Ye H. L. Cheng D. Zhang G. T. Zhou J. D. (2018). Upregulation of LncDQ is associated with poor prognosis and promotes tumor progression via epigenetic regulation of the EMT pathway in HCC. *Cell. Physiol. Biochem.* 46 1122–1133. 10.1159/000488841 29669339

[B39] Zhang Z. Deng X. Liu Y. Liu Y. Sun L. Chen F. (2019). PKM2, function and expression and regulation. *Cell. Biosci.* 9:52. 10.1186/s13578-019-0317-8 PMC6595688 31391918

[B40] Zou J. Huang R. Li H. Wang B. Chen Y. Chen S. (2019). Secreted TGF-beta-induced protein promotes aggressive progression in bladder cancer cells. *Cancer Manag. Res.* 11 6995–7006. 10.2147/cmar.s208984 31440088 PMC6664251

