# Supplementary figures and images for: Dihydropyrimidinase Like 2 Promotes Bladder Cancer Progression via Pyruvate Kinase M2-Induced Aerobic Glycolysis and Epithelial–Mesenchymal Transition
Source: Front Cell Dev Biol. 2021 Jul 6;9:641432. doi: 10.3389/fcell.2021.641432 (PMC8291048; doi:10.3389/fcell.2021.641432)

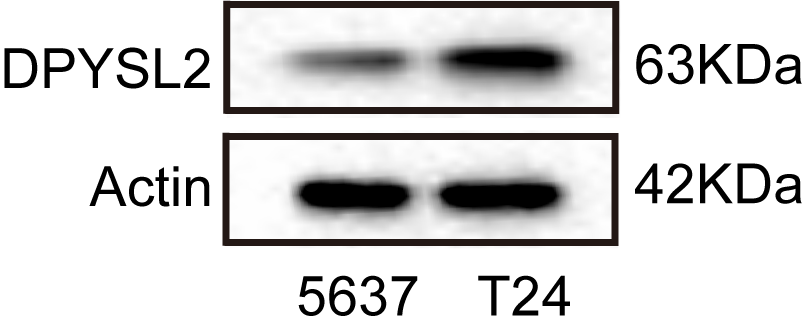

Supplement: Supplementary file 1 [file Image_1.TIF]

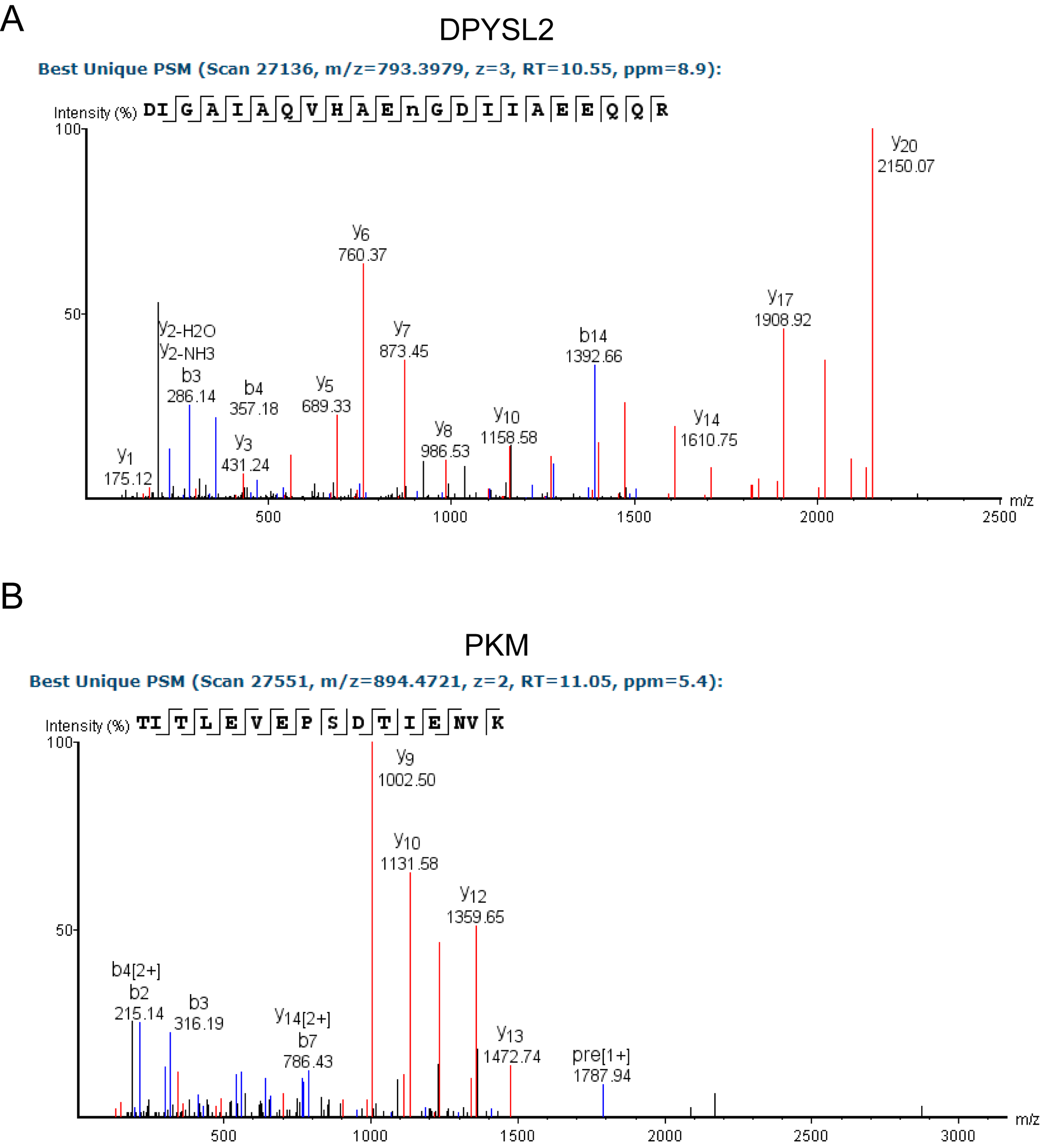

Supplement: Supplementary file 2 [file Image_2.TIF]

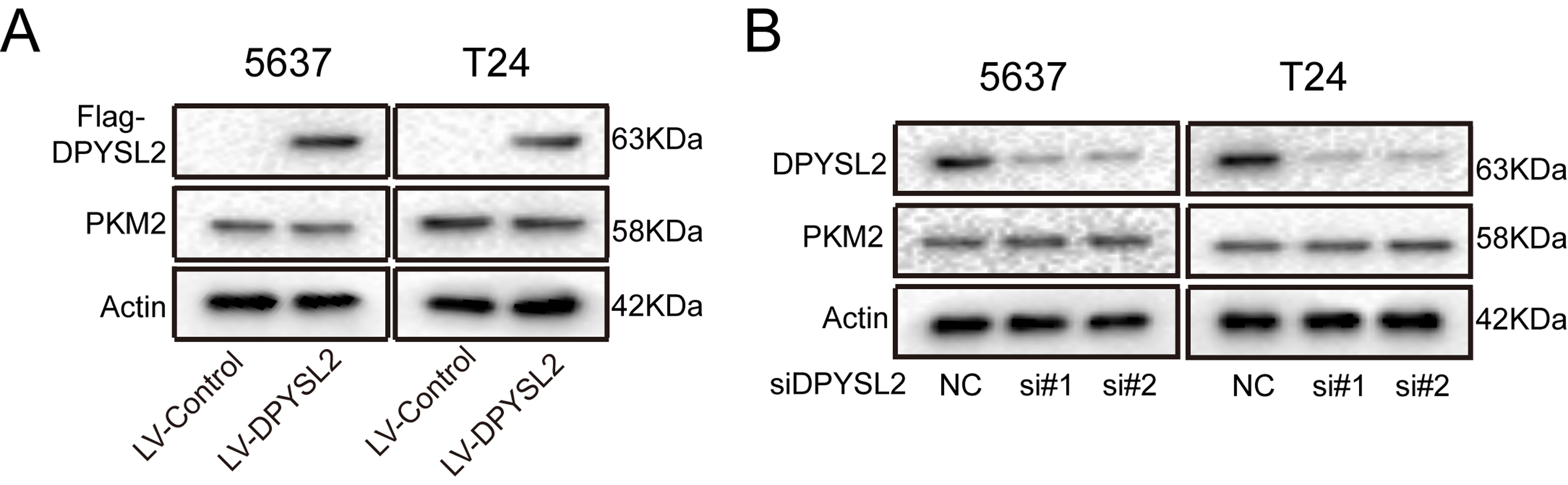

Supplement: Supplementary file 3 [file Image_3.TIF]

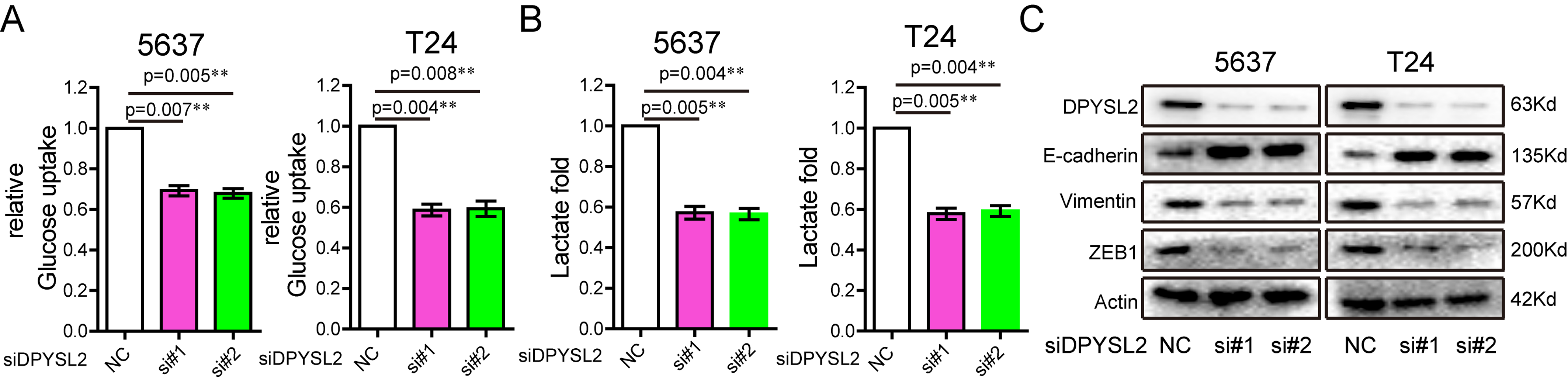

Supplement: Supplementary file 4 [file Image_4.TIF]
