# Supplementary material for: Dihydropyrimidinase Like 2 Promotes Bladder Cancer Progression via Pyruvate Kinase M2-Induced Aerobic Glycolysis and Epithelial–Mesenchymal Transition
Source: Front Cell Dev Biol. 2021 Jul 6;9:641432. doi: 10.3389/fcell.2021.641432 (PMC8291048; doi:10.3389/fcell.2021.641432)
Supplement: Supplementary file 6 [file Data_Sheet_1.DOCX]

**Supplemental figure legends**

**Figure S1.** The basal level of DPYSL2 in 5637 and T24 cells was detected using western blot.

**Figure S2.** DPYSL2 and PKM were identified using mass spectrometry. (A) Unique peptides in the DPYSL2 protein were identified by mass spectrometry. (B) Unique peptides in the PKM protein were identified by mass spectrometry.

**Figure S3. (A)** The protein expression levels of Flag-DPYSL2 and PKM2 were detected in 5637 and T24 cell lines stably overexpressing DPYSL2. (B) Anti-DPYSL2 siRNAs were transfected into 5637 and T24 cells. The protein expression levels of DPYSL2 and PKM2 were measured.

**Figure S4.** Anti-*DPYSL2* siRNAs were transfected into 5637 and T24 cells, Glucose uptake (A), lactate production (B), and levels of EMT markers (C) were measured.
